# Chronic diseases attributable to a diet rich in processed meat in Brazil: Burden and financial impact on the healthcare system

**DOI:** 10.3389/fnut.2023.1114766

**Published:** 2023-03-15

**Authors:** Carla Eduarda Faustino Rocha, Magda do Carmo Parajára, Ísis Eloah Machado, Aline Siqueira Fogal Vegi, Mariana Carvalho de Menezes, Adriana Lúcia Meireles

**Affiliations:** ^1^Postgraduate Program in Health and Nutrition, School of Nutrition, Federal University of Ouro Preto, Ouro Preto, Brazil; ^2^Department of Family Medicine, Mental and Collective Health, School of Medicine, Federal University of Ouro Preto, Ouro Preto, Brazil; ^3^Department of Clinical and Social Nutrition, School of Nutrition, Federal University of Ouro Preto, Ouro Preto, Brazil

**Keywords:** burden of disease, meat products, premature death, costs and cost analysis, diet

## Abstract

**Background:**

The consumption of processed meat causes negative impacts on health; however, this burden for the population living in developing countries is less explored. This study aimed to describe the burden of chronic noncommunicable diseases (NCDs) attributed to a diet rich in processed meat between 1990 and 2019 in Brazil and its federative units and the financial burden on the Unified Health System (SUS) in 2019.

**Methods:**

Secondary data from the Global Burden of Disease (GBD) and SUS Information Systems were used in this ecological study. The metrics to assess the burden of NCDs attributable to processed meat consumption were disability-adjusted life years (DALYs) and deaths. The age-standardized rates were presented per 100,000 inhabitants with 95% uncertainty intervals (95% UI). The cost of hospitalizations and outpatient procedures covered by SUS for the treatment of NCDs attributable to processed meat consumption was estimated using the population-attributable fraction. Both burdens were estimated for both sex and stratified by sex, specific cause, and federative units.

**Results:**

The age-standardized DALY rates attributable to a diet rich in processed meat increased between 1990 (75.31/100,000 [95% UI: 34.92–139.65]) and 2019 (79.35/100,000 [95% UI: 42.84–126.25]); while mortality rates remained stable between 1990 (2.64/100,000 [95% UI: 1.17–5.21) and 2019 (2.36/100,000 [95% UI: 1.22–4.09]). The cost of hospitalization and outpatient procedures in Brazil for NCDs attributable to the consumption of processed meat was approximately US$ 9,4 million, of which US$ 6,1 million was spent on ischemic heart disease, US$ 3,1 million on colorectal cancer, and US$ 200 thousand on type 2 diabetes mellitus.

**Conclusion:**

The NCD burden did not decrease during the years evaluated, while the financial burden was high in 2019, with higher treatment costs for ischemic heart disease. These results can guide political, economic, and health education interventions to advance the fight against NCDs.

## 1. Introduction

Chronic noncommunicable diseases (NCDs) are responsible for seven of the top ten causes of death worldwide, according to the 2019 Global Health Estimates (1). In Brazil, NCDs represent 74% of causes of death and approximately 30% of all NCD-related deaths are classified as premature (30–69 years), with an emphasis on cardiovascular diseases, chronic respiratory diseases, neoplasms, and diabetes mellitus (2).

Food consumption in the western population has been reported as a potential risk factor for the emergence of NCDs (1–3). This consumption is often characterized by a high intake of refined cereals, processed and ultra-processed foods, and red meats; insufficient consumption of fruits and vegetables; and low fiber, vitamin, and mineral content (4, 5). Specifically, high consumption of red and processed meats is a relevant risk factor for increased risk of NCDs disease and mortality (6).

Global meat consumption has increased significantly over the last decades. To illustrate this scenario, the world’s production rose from 70 million tons in 1960 to 315 tons in 2013, with red and processed meat representing over 60% of this total (7). Most recently, in 2019, world meat production reached 339 million tons (8). Although excessive consumption of red meat is considered harmful to the environment and health, limited consumption is recommended since it is a dietary supply of nutrients such as proteins, iron, potassium, zinc, selenium, riboflavin, niacin, thiamine, and vitamin B12; however, there is no recommendation for processed meat (9).

Processed meat is any type of meat that has been transformed by salting, curing, fermentation, smoking, and other processes to enhance the flavor or improve preservation, such as ham, sausage, bacon, salami, bologna, and turkey blanquet (10). Due to these characteristics, processed meats can contain high concentrations of fat, especially saturated fat, and sodium, in addition to compounds such as polycyclic aromatic hydrocarbons and preservatives such as nitrites and nitrates, which have been related to the emergence of cardiovascular diseases, diabetes mellitus, and gastrointestinal tract cancer (11).

Although processed meat seems to have a higher risk of causing disease in high-income countries (12), the disease burden caused by this risk factor in low- and middle-income countries is less known. In addition to the disease burden attributable to diet, a financial burden also exists, particularly for treating these NCDs by the health system (13). In Brazil, one of the largest meat producers in the world (8), only a few studies have investigated the burden and costs of diseases attributed to dietary risk factors, especially the consumption of processed meat. This knowledge can help evaluate the impact of processed meats on health and subsidy the balanced and efficient allocation of public budgets in a middle-income country, assisting in the decision-making process for public health policies (13).

In this context, this study aimed to analyze the disability-adjusted life years (DALYs) and deaths of NCDs attributed to a diet rich in processed meat in Brazil, between 1990 and 2019, and determine the financial burden on the Brazilian Unified Health System (SUS) in 2019.

## 2. Methods

### 2.1. Study design and data sources

This descriptive ecological study used the secondary data from the Global Burden of Disease (GBD) Study 2019 conducted by the Institute for Health Metrics and Evaluation (IHME) and data from the SUS Hospital and Outpatient Information Systems from the Brazilian Health Ministry. Both disease and financial burden attributable to the diet rich in processed meat in Brazil and its 27 federative units (FUs, also referred to as states) were obtained for the population aged 25 years or over.

The GBD is a relevant data source for science and public health to estimate the mortality and disabilities attributable to several risk factors, including inadequate diet (14). The GBD seeks to subsidy the development of public policies to reduce the disease burden worldwide. To produce estimates capable of quantifying and comparing the magnitude of health loss resulting from diseases, injuries, and risk factors by age and sex in different geographic regions over time (14, 15), the GBD study uses data from multiple sources from 195 countries, such as vital statistics, population censuses, administrative databases, scientific publications, surveys, cancer registries, and environmental and occupational data (14, 15). All estimates produced by this study are publicly available at: http://ghdx.healthdata.org/gbd-results-tool. The data used in this study were retrieved on February 4, 2021.

Data on direct financial costs (such as medical consultations, hospitalizations, medications, tests, inputs, emergencies, and other services related to the patient care) were collected through Outpatient Information System (SIA/SUS) and Hospital Information System (SIH/SUS). The SIA/SUS is a platform used to register outpatient production, following the logic of payment per procedure. The data collection instruments used to extract data from the SIA/SUS were the Outpatient Production Bulletin (BPA), which includes the monthly record of all procedures performed, and the Authorization of High Complexity Procedures (APAC), used in charging, authorization, and provision of epidemiological information (16). The SIH/SUS is a platform for registering hospitalizations which uses the Hospital Admission Authorization (AIH) issued after analyzing the hospitalization as an instrument to collect the data (16). All these cost data were obtained at the Department of Informatics of the Unified Health System (DATASUS), publicly available on the website http://datasus.saude.gov.br/, and retrieved on May 26, 2022.

### 2.2. Burden of disease

The GBD estimates the burden of disease attributable to the risk factors using a comparative risk assessment referred as population attributable fraction (PAF), which represents the proportion of the disease burden that could be avoided if theoretical exposure to the risk factor (processed meats) was minimal in the past (14). To estimate PAF, the following data are necessary: the exposure level (where l is the highest limit, and u is the lowest consumption limit) for the dietary risk (P), the relative risk (RR) obtained after the survey of systematic reviews and meta-analyses that show epidemiological evidence of the causal relationship between the health outcome and the consumption of processed meat, and the level of exposure related to the lowest risk of developing a certain health outcome (theoretical minimum level of risk exposure, TMREL) (14). This information allows the estimation of PAF for each risk-disease pair by age (a), sex (s), country (c), and year (t), using the formula described below:


$${\text{PAF}}_{\text{asct}}=\frac{\int_{l}^{u}{\text{RR}}_{\text{as}}\left(x\right)P_{\text{asct}}\left(x\right)dx-{\text{RR}}_{\text{as}}\left(\text{TMREL}\right)}{\int_{l}^{u}{\text{RR}}_{\text{as}}\left(x\right)P_{\text{asct}}\left(x\right)dx}.$$

To estimate the level of exposure to a diet rich in processed meat in Brazil, the GBD 2019 study used input data from the Health Survey conducted in the Municipality of São Paulo in 2008, data from the Family Budget Survey in 2008–2009, and data on the commercialization of processed meats made available by the Ministry of Health and Euromonitor International (17). Since the data are obtained from different sources that use different methods, a network meta-regression was used to adjust the data and make them comparable with those obtained using the standard reference method of consumption assessment, the 24-h dietary recall. After standardization, the estimates of consumption per day, person, sex, age group, location, and year were determined using a Gaussian spatiotemporal regression model (14).

The level of exposure related to a lower risk of developing unfavorable health outcomes (the TMREL) was considered equal to 0 g/day, as reported by the GBD 2019 study (1). Thus, the concept of a diet rich in processed meats used in the present study corresponds to the daily consumption of any amount of cured, smoked, salted meats, or with the addition of chemical preservatives (18).

In the GBD 2019 study, the outcomes associated with a diet rich in processed meats included ischemic heart disease, type 2 diabetes mellitus, and colorectal cancer. By convention, the GBD study calculated the burden attributable to dietary risks only for the population aged 25 years (14).

The total number of deaths or DALYs attributable to all relevant outcomes (w) were calculated using data from disease-specific (o) and disease-specific PAFs. DALY and mortality rates were calculated according to the following formula:


$$\text{Total attributable DALYs or deaths}=\sum_{o=1}^{w}\text{DALYs}\;{\text{or deaths}}_{\text{oasct}}x\;{\text{PAF}}_{\text{oasct}}.$$

A previous publication described the methodology used to obtain the number of DALYs and deaths for each cause of death and disability in the GBD study (15) and the attributable burden of disease (14).

The present study presents a time series with standardized DALY rates and mortality of NCDs attributed to a diet rich in processed meat in Brazil between 1990 and 2019. The findings were shown for both sexes together and stratified by sex. Maps with the distribution of NCD DALY rates and mortality attributed to a diet rich in processed meat in the FUs in 1990 and 2019 were built in QGIS version 3.22.7. Also, a description of the DALY rates and mortality for the specific causes of NCDs attributable to a diet rich in processed meat in 2019 was presented.

All rates were expressed as age-standardized rates per 100,000 inhabitants using the GBD world population as a reference for both sexes. The results of the burden of disease attributed to the consumption of processed meat were expressed as 95% uncertainty intervals (95% UIs) and aimed to incorporate the uncertainty of the parameters (14).

### 2.3. Financial burden

The cost of hospitalization and outpatient procedures covered by SUS for the treatment of NCDs attributed to the consumption of processed meats was estimated using the top-down approach. The cost data were extracted from SIA/SUS and SIH/SUS using the Microdatasus package for the R statistical program, which allows direct extraction from the DATASUS server and subsequent processing of these data (19). These databases were filtered to present only the costs related to hospitalizations and procedures performed in 2019. Subsequently, the STATA software version 15 was used to group the outcomes from hospitalizations and procedures and the GBD 2019 causes attributable to a diet rich in processed meat through the International Classification of Diseases 10th Revision (ICD-10) codes. Then, Microsoft Excel was used to link the PAF values from GBD 2019 and the costs, by age, sex, specific cause, and FUs.

The costs were calculated by multiplying the PAF provided by the GBD 2019 study with the amounts spent by the SIA/SUS and SIH/SUS, which present the diagnosis related to each procedure performed and the amounts actually paid by the SUS. Cost data were stratified by type of procedure (outpatient or hospitalization), sex, specific cause, and FU. In addition, to compare the results by FU, the total cost in the FU was divided by its resident population (data retrieved from DATASUS) and multiplied by 10,000 inhabitants. The values were obtained in reais (R$) and converted into dollars (US$), the latter being estimated according to the quotation provided on December 31, 2019 (US$ 1 = R$ 3,944) (20).

## 3. Results

**Figure 1:**
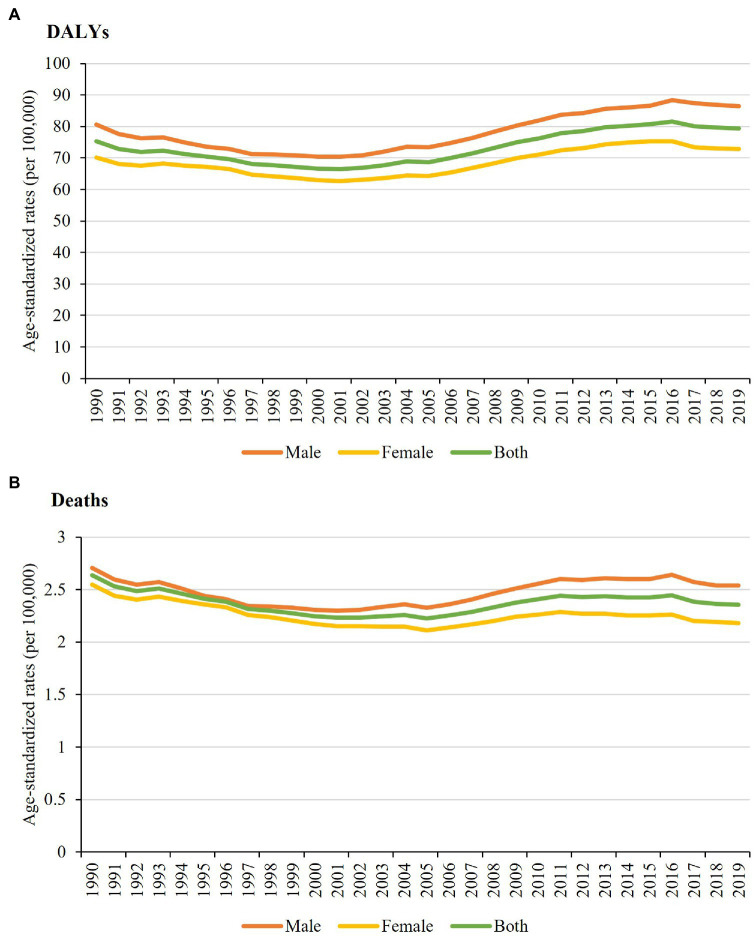
Age-standardized disability-adjusted life years **(A)** and deaths **(B)** rates per 100,000 inhabitants for chronic noncommunicable diseases attributable to a diet rich in processed meat for both sexes and stratified by sex in Brazil between 1990 and 2019.

Figure 1 illustrates the standardized DALY rates and mortality of NCDs attributed to a diet rich in processed meat in Brazil between 1990 and 2019 for both sexes pooled and stratified by sex in Brazil. Regarding the age-standardized DALY rates for both sexes, a slight increase was observed over the period: 75.31/100,000 inhabitants (95% UI: 34.92–139.65) in 1990 and 79.35/100,000 inhabitants (95% UI: 42.84–126.25) in 2019, with a declining trend between 1997 and 2005. Through the study period, the age-standardized mortality rates among men were higher than those among women (Figure 1A; Supplementary Table S1).

From 1990 to 2005, the age-standardized mortality rates for NCDs attributable to a diet rich in processed meat for both sexes showed a low variation, with rates of 2.64/100,000 inhabitants (95% UI: 1.17–5.21) in 1990 and 2.22/100,000 inhabitants (95% UI: 1.05–4.01) in 2005. Subsequently, the rate increased (2.36/100,000 inhabitants; 95% UI: 1.22–4.09) and reached closer to the initial level in 2019. From 2001 onward, the age-standardized mortality rates for men and women began to differ, with rates for men being slightly higher (Figure 1B; Supplementary Table S2).

**Figure 2:**
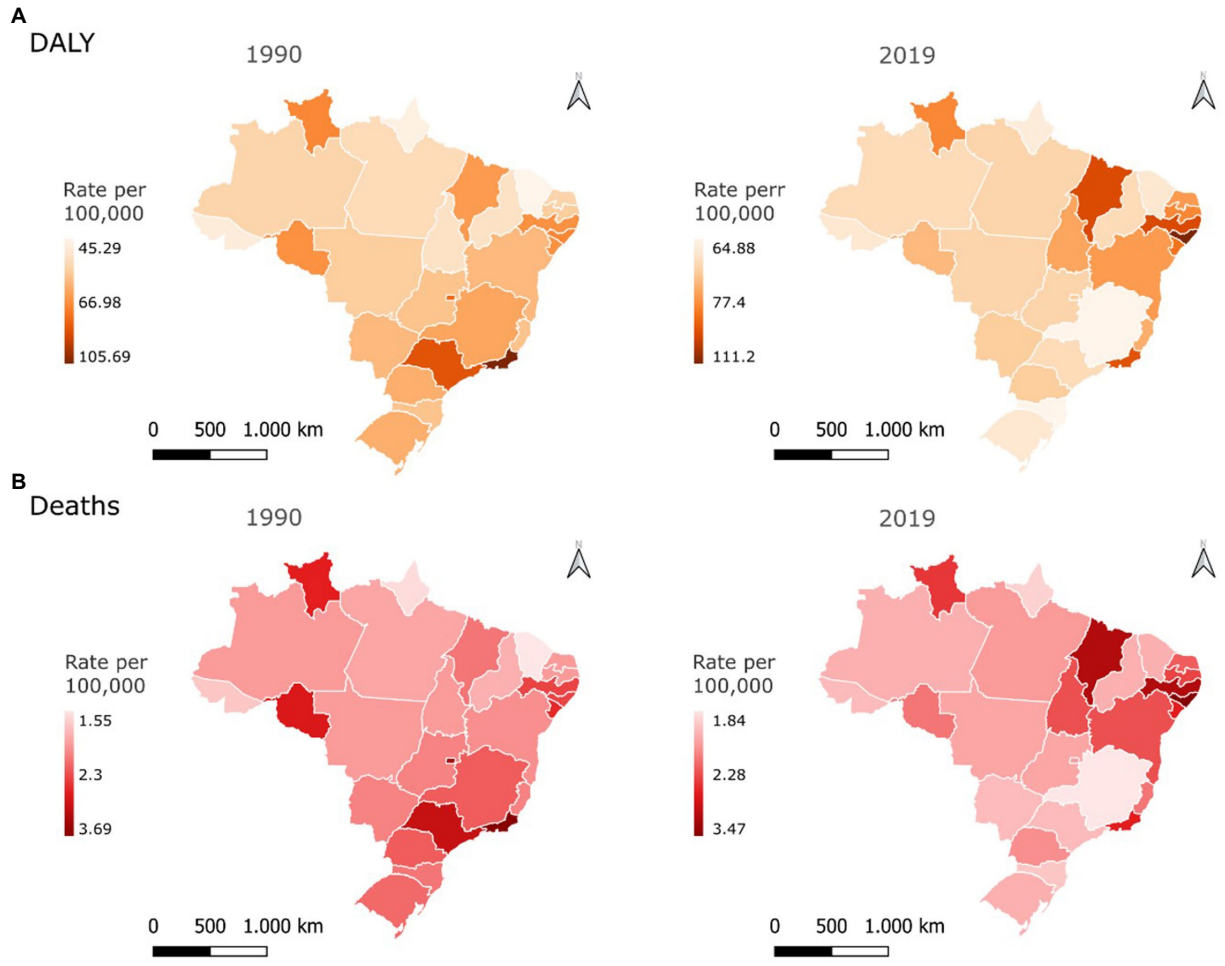
Age-standardized disability–adjusted life years **(A)** and deaths **(B)** rates per 100,000 inhabitants for chronic noncommunicable diseases attributable to a diet rich in processed meat for both sexes in the federative units of Brazil in 1990 and 2019.

Figure 2 shows the age-standardized DALY rates and mortality of NCDs attributable to a diet rich in processed meat for both sexes in the Brazilian FUs in 1990 and 2019. In 1990, the states of the Southeast and South regions had the highest rates of DALYs, especially Rio de Janeiro (105.69/100,000 inhabitants; 95% UI: 48.96–201.30) and São Paulo (88.55/100,000 inhabitants; 95% UI: 40.66–170.26; Figure 2A; Supplementary Table S3). Regarding age-standardized DALY rates, the Northeast states presented higher rates in 2019, with emphasis on the state of Alagoas (111.20/100,000 inhabitants; 95% UI: 58.26–173.60), while the Southeast states had the lowest rates, with emphasis on the state of Minas Gerais (64.88/100,000; 95% UI: 34.03–106.81; Figure 2A; Supplementary Table S3).

In 1990, the mortality rates in the states of the South and Southeast were higher, especially in Rio de Janeiro (3.69/100,000 inhabitants; 95% UI: 1.59–7.34) and São Paulo (3.17/100,000 inhabitants; 95% UI: 1.34–6.65). Meanwhile, the states in the Northeast and North regions had the lowest rates, especially in Ceará (1.55/100,000 inhabitants; 95% UI: 0.67–2.99) and Amapá (1.66/100,000 inhabitants; 95% UI: 0.73–3.28; Figure 2B; Supplementary Table S3). The uncertainty intervals were relatively large. In 2019, the northeastern states had the highest mortality rates, especially the state of Alagoas (3.47/100,000 inhabitants; 95% UI: 1.76–5.64). By contrast, the Southeast states had the lowest mortality rates in 2019, especially the state of Minas Gerais (1.84/100,000 inhabitants; 95% UI: 0.92–3.28; Figure 2B; Supplementary Table S3).

**Figure 3:**
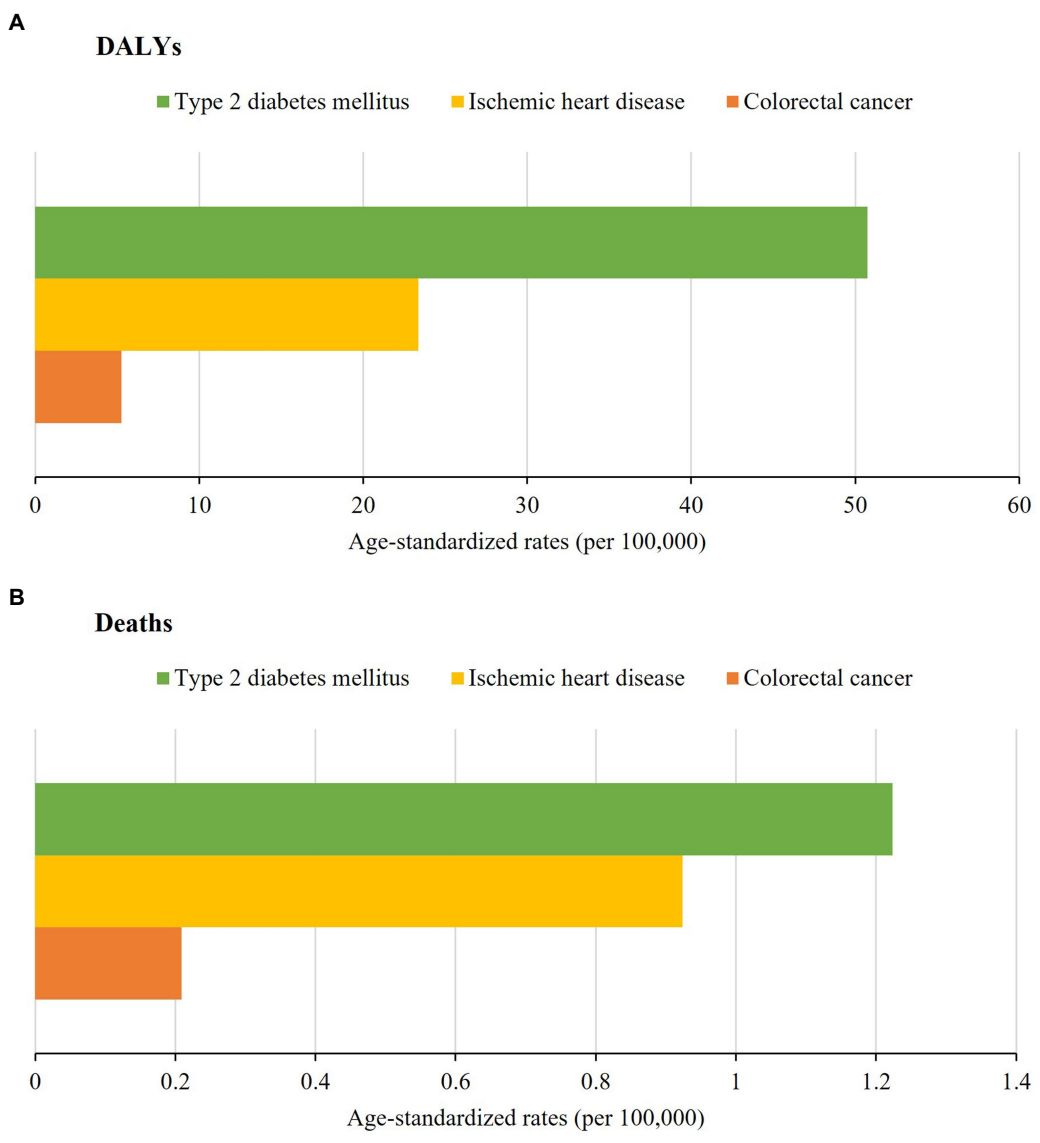
Age-standardized disability-adjusted life years **(A)** and deaths **(B)** rates per 100,000 inhabitants according to specific causes of chronic noncommunicable diseases attributable to a diet rich in processed meat for both sexes in Brazil in 2019.

Figure 3 presents the age-standardized rates of specific causes of DALYs and deaths attributable to a diet rich in processed meat in both sexes in Brazil in 2019. Type 2 diabetes mellitus contributed to the highest age-standardized DALY rate (50.75/100,000 inhabitants; 95% UI: 28.29–67.27), followed by ischemic heart disease (23.35/100,000 inhabitants; 95% UI: 3.35–67.43) and colorectal cancer (5.26/100,000 inhabitants; 95% UI: 0.67–9.05; Figure 3A; Supplementary Table S4). The highest mortality rate was also due to type 2 diabetes mellitus (1.22/100,000 inhabitants; 95% UI: 0.67–1.52), followed by ischemic heart disease (0.92/100,000 inhabitants; 95% UI: 0.16–2.66) and colorectal cancer (0.21/100,000 inhabitants; 95% UI: 0.03–0.36; Figure 3B; Supplementary Table S4).

The costs of hospitalizations and outpatient procedures of NCDs attributable to a diet rich in processed meat for the SUS in 2019 are shown in Table 1. When the costs of hospitalization and outpatient procedures performed in the public health network were summed up, Brazil spent US$ 9,383,944.43. Of the amount, 73.9% (US$ 6,930,837.62) were spent on hospitalizations, with US$ 4,119,830.10 and US$ 2,811,007.52 spent on male and female patients, respectively. For outpatient costs, Brazil spent US$ 2,453,106.81(26.1%), of which US$ 1,140,330.26 and US$ 1,312,776.55 were spent on male and female patients, respectively. Of the total costs of NCDs attributed to a diet rich in processed meat in Brazil, 64.8% (US$ 6,077,897.82) corresponded to the treatment for ischemic heart disease. Approximately 33.5% (US$ 3,145,390.55) was spent on colorectal cancer treatment. Type 2 diabetes mellitus corresponded to 1.7% (US$ 160,656.06).

**Table 1:** Total costs of hospitalizations and outpatient procedures for chronic noncommunicable diseases attributable to a diet rich in processed meat to the Unified Health System in Brazil in 2019.

Outcome	Hospitalization costs in US$		Outpatient care costs in US$		Total
	Male	Female	Male	Female	
Ischemic heart disease	3,504,673.15	2,099,622.13	228,533.12	245,069.42	**6,077,897.82**
Type 2 diabetes mellitus	75,732.16	75,411.79	4,451.51	5,060.60	**160,656.06**
Colorectal cancer	539,424.79	635,973.60	907,345.63	1,062,646.53	**3,145,390.55**
**Total**	**4,119,830.10**	**2,811,007.52**	**1,140,330.26**	**1,312,776.55**	**9,383,944.43**

The bold values represent the total costs to the Unified Health System in Brazil and the costs stratified by the outcome and by sex and type of procedure.

**Figure 4:**
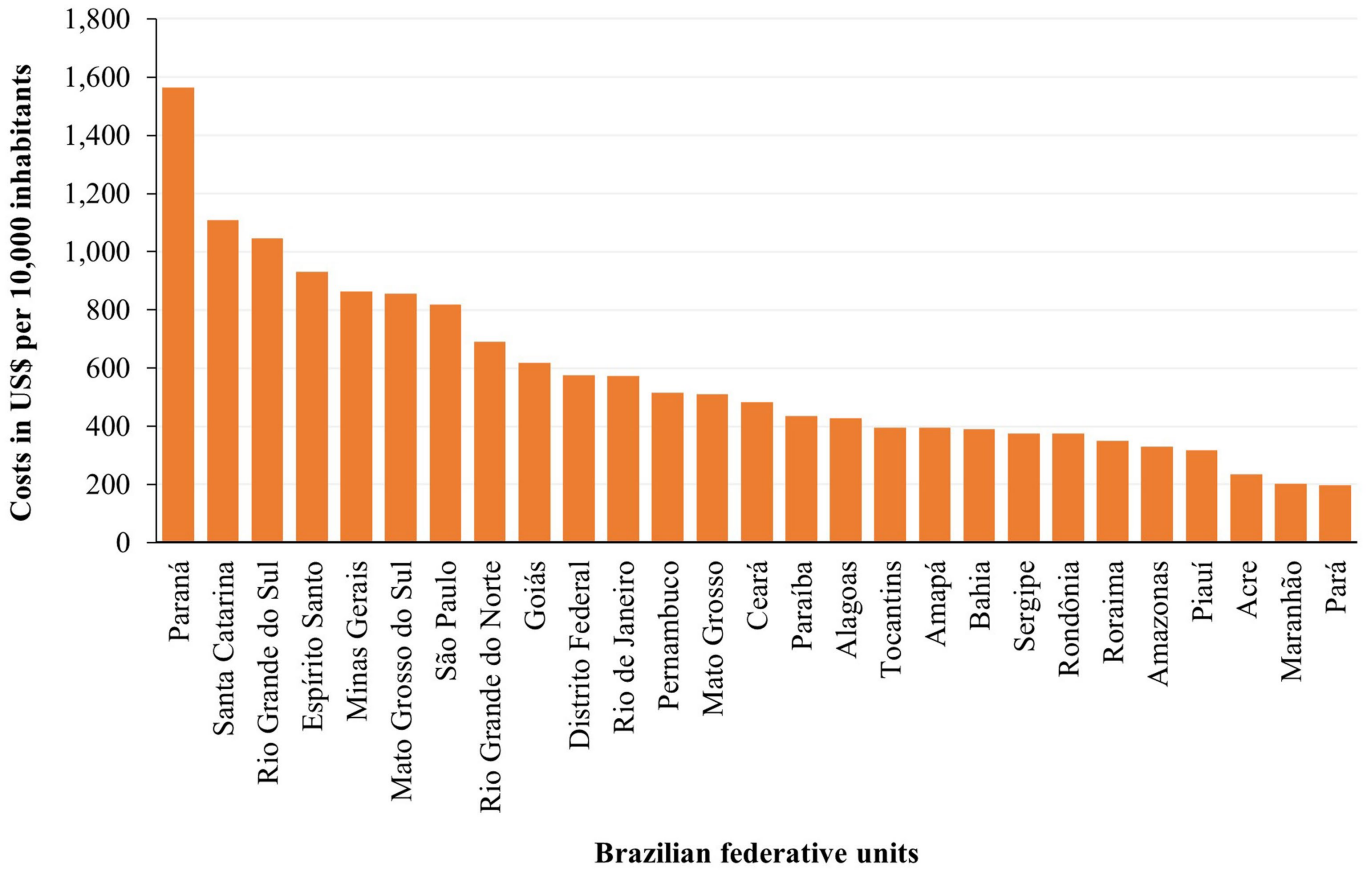
Costs of hospitalizations and outpatient procedures per 10,000 inhabitants for chronic noncommunicable diseases attributable to a diet rich in processed meat to the Unified Health System in the Brazilian federative units in 2019.

The costs per 10,000 inhabitants of hospitalizations and outpatient procedures of NCDs attributed to a diet rich in processed meat for the SUS in 2019, stratified by Brazilian FU, are illustrated in Figure 4 (Supplementary Tables S5, S6). The costs were higher in the states of Paraná (US$ 1,564.06), Santa Catarina (US$ 1,107.59), and Rio Grande do Sul (US$ 1,046.32), the three states from the South region. The Pará, Maranhão, and Acre, which are states located in North and Northeast regions, had the lowest *per capita* costs (US$ 167.57, US$ 203.50, and US$ 235.79, respectively).

## 4. Discussion

The burden of NCDs attributable to a diet rich in processed meat declined between 1990 and 2005 in Brazil; however, from that year onward, the age-standardized DALY rates and mortality started to increase again until 2019. Geographically, this burden eventually changed over the study period, shifting the highest burden in the Southeast states in 1990 to the Northeast states in 2019. In addition to the increased health burden of a diet rich in processed meat, especially type 2 diabetes mellitus, the costs of managing NCDs in 2019 were high. These costs were higher for hospitalizations among men due to the treatment of ischemic heart disease and for those residing in the South region.

Over the last three decades, the burden of NCDs attributable to a diet rich in processed meat remained stable or slightly increased in Brazil. Despite some substantial decline in the DALY rate and mortality over time, the rates presented an increased trend after 2005 in the country. In line with this, a study investigating the burden of NCDs attributable to 15 dietary risk factors in Brazil showed the higher participation of processed meat consumption as a contributor for diseases attributable to dietary risk factors from 1990 (12ª position) to 2019 (10ª position) (21). This trend might reflect the high consumption of processed meat in the Brazilian population (15 g/day), representing 12% of the total meat intake (22). The World Cancer Research Fund (WCRF) recommends the consumption of no more than 300 g (cooked weight)/week of red meat, very little to be processed (11), which suggests that the consumption of processed meat is much more than the WRCF recommendation.

The estimates in the GBD 2019, considering the worldwide scenario, do not corroborate our findings. At the global level, a decrease was observed in age-standardized DALYs (1990: 145.57/100,000 [95% UI: 63.48–226.67]; 2019: 104.35/100,000 [95% UI: 64.34–154.35]) and deaths (1990: 6.6/100,000 [95% UI: 2.34–10.97]; 2019: 3.9/100,000 [95% UI: 1.96–6.25]) of NCDs attributable to a diet rich in processed meat (23). Contrary to the worldwide decreasing trend, similar trends and findings to Brazil was found in Latin America and the Caribbean to the age-standardized NCDs rates related to the consumption of processed meats, that is, stability or slight increase in these rates over time: DALYs, 1990: 76.42/100,000 (95% UI: 38.26–128.52), 2019: 82.91/100,000 (95% UI: 45.75–127.87); deaths, 1990: 2.6/100,000 (95% UI: 1,27–4.73), 2019: 2.44/100,000 (95% UI: 1,28–4.04) (23).

Our results reinforce the need for interventions to reduce the disease burden attributable to processed meat in low- and middle-income countries. Processed meat consumption was expected to be even higher in regions with better socioeconomic positions (9) compared to countries in Latin America and the Caribbean, such as Brazil. A possible explanation for the increasing trend in disease burden in low- and middle-income countries is the reduced improvements in treatment and emergency services over time, besides the increased consumption of processed meat in the last years (13). In addition, our finding emphasizes the importance of investigating specific realities from populations all over the world since could be expected a higher risk of disease attributable to this dietary risk factor in the high-income populations (12).

Thus, the disease burden also differs according to socioeconomic status, with low-income populations most affected by the impact of this attributable burden (14). The most recent results in our study showed a higher financial cost of NCDs attributable to a diet rich in processed meat in states from the South region. The sociodemographic and cultural differences among the Brazilian regions may justify a high consumption of processed meats in the South region and more income to invest in procedures to recuperate the population health in this region (24, 25).

The burden of NCDs caused by processed meat consumption was higher among men. A reliable explanation is a concern about healthier choices by women and the higher consumption of meat among men, also considered a cultural factor, symbolizing strength and power (12, 25).

In 2019, the prevalence of type 2 diabetes mellitus, ischemic heart disease, and colorectal cancer was equal to 5.76% (95% UI: 5.23%–6.30), 1.92% (95% UI: 1.65–2.25%), and 0.09% (95% UI: 0.08–0.09%), respectively (23). Although there is a commitment to reducing NCDs through prevention and treatment in the country, the implementation of current solutions has been insufficient to effectively control this group of diseases and achieve the third goal of sustainable development, which consists of reducing by one-third premature mortality from NCDs by 2030 (1).

In 2019 in Brazil, the outpatient costs associated with NCDs attributed to a diet rich in processed meats were higher among women as compared to men, and the opposite was true for hospitalization costs. Between 1998 and 2013, Brazilian women were approximately 70% more likely to have NCDs than men, and the risk increased with advancing age in women (26). With the estimated increase in life expectancy especially in women (80.25 years in 2020 and 81.22 years in 2025) (27), the treatment of multiple chronic diseases will generate additional healthcare-related expenses (28). A hypothesis that justifies the higher costs of outpatient procedures in women and hospitalization in men is that women are more health conscious and seek healthcare services more frequently, especially outpatient procedures (29).

Type 2 diabetes mellitus was the primary cause of mortality and DALYs related to a diet rich in processed meat in the country, but SUS spent more in terms of the treatment of ischemic heart disease and colorectal cancer. Notably, this study only included specialized procedures and hospitalizations, and a high proportion of treatments for diabetes mellitus is performed in primary health care hospitals. The consumption of processed meats contributes to the increase in the mortality rate because nitrites and nitrates are widely used as additives in processed meats and can be converted into nitrosamines, which are toxic to the pancreatic β cells and are associated with insulin resistance (30). In terms of the economic burden, diabetes mellitus and its complications contributed to approximately 12% of global health expenditures in 2017, making it necessary to adopt effective strategies to reduce the global health and economic burden related to diabetes mellitus (31). Brazil is one of the countries worldwide with the highest incidence of diabetes mellitus and has the third most expensive healthcare bill associated with the treatment of this condition (US$ 52.3 billion in 2019) (32), thus highlighting the importance of this disease in terms of healthcare expenditures.

Ischemic heart disease attributed to the consumption of processed meat was the second most common cause of the burden of NCDs in Brazil in 2019, which had the highest budget allocated by the SUS. According to the World Health Organization, in 2019, 85% of premature deaths were caused by cardiovascular diseases in low- and middle-income countries (1). Direct costs related to the management of cardiovascular diseases in Brazil have a negative impact on the country’s budget, with an emphasis on the acquisition of medicines, hospitalizations, and the levels of tertiary or quaternary care, which included high-complexity treatment (33, 34).

Colorectal cancer attributed to the consumption of processed meats was the third highest contributor to the burden of NCDs and healthcare costs in 2019, having the second most expensive budget allocated by the SUS. The International Agency for Research on Cancer stated that existing categorical evidence support the classification of processed meat as carcinogenic to humans (35). In Brazil, estimates reported in 2018 showed that colorectal cancer was the third most common cancer type in men (17,380 cases; 8.7%), followed by women (18,980 cases; 9.4%) (36). According to the Ministry of Health, in 2010–2015, the expenses related to cancer care by SUS increased by approximately 70% (37). In addition to the social and health burden, the economic burden of cancer is relatively substantial in Brazil and tends to increase in the near future, as a reflection of improved access to healthcare and increasing chances of survival, population growth, and aging (38).

Increased consumption of processed meat contributes to an increase in the attributable burden of diet related NCDs in developed and developing countries (39). Brazil is the second largest producer of beef (40) and the largest exporter among the countries worldwide, exporting a fifth of its total production, and this sector is one of the main drivers of deforestation (41). In addition, there is an urgent need to control animal protein consumption as a sustainable development strategy considering that meat production is among the main factors responsible for global warming and environmental degradation (40).

Robust analysis studies with modeling have indicated the potential benefits of changes in dietary patterns, emphasizing the necessary decrease in current meat consumption to reduce the use of environmental resources and its positive effect on the risk of premature mortality associated with dietary risk factors and costs of living (42, 43). Therefore, the consumption of adequate and healthy food should not only be viewed as a human right of everyone, but also as a matter of maintaining environmental balance, since it involves personal and public health, as well as global environmental sustainability (40).

A diet rich in processed meat is considered an avoidable risk factor since the reduction of this consumption is possible and desirable to promote through prevention and health promotion. Deaths in populations with the greatest risk can also be prevented (44). Taxation, food and nutrition education, and provision of warning labels on consumption are alternatives to disincentivizing the consumption of processed meat, but political viability is influenced by the economic and political power of the meat industries (45).

The food guide for the Brazilian population is a fundamental tool to discourage the consumption of ultra-processed foods, including processed meats (46, 47). A greater consumption of ultra-processed foods is associated with a decrease in the quality of the diet and a relatively increased risk of all causes of premature death, given that these foods have high energy load and low micronutrient content in addition to chemical additives, which negatively impacts the quality of the diet (48, 49).

This study has some limitations. The plurality of Brazilian regions can interfere with the quality of the database due, for example, to under-registration or registration of wrong or missing information. The disease burden data were reviewed and corrected by GBD; however, the corrections were not applied to the cost data. Further, the results of more current national food surveys, such as Family Budget Survey, were not included in the estimate. Moreover, the SUS database only reported the total amount reimbursed by the federal government to the country’s healthcare system, which does not consider other types of expenditure by states and municipalities.

## 5. Conclusion

A stable trend or slight increase of NCDs attributable to a diet rich in processed meat was observed in Brazil between 1990 and 2019. Most recently, the greatest NCD burden is shown in Northeast states. In addition to the high health burden of the diet rich in processed meat, the health expenditures of the country for the treatment of NCDs in 2019 were high, with these costs being higher among men due to treatment of ischemic heart disease, and for the population residing in the South region.

Our results reveal the urgency of controlling this risk factor to protect the health of the population and enable diversification of financial investments. The adoption of public policies should focus on the taxation of processed meats and implementation of food and nutrition education strategies, thus reinforcing the revision of the food guide for the Brazilian population, are possible alternatives that can be adopted to reduce the burden and costs of NCDs that are attributable to dietary habits considered as risky eating behaviors.

## Data availability statement

The original contributions presented in the study are included in the article/Supplementary material, further inquiries can be directed to the corresponding author/s.

## Ethics statement

This study was exempted from evaluation by the Research Ethics Committee of the Federal University of Ouro Preto (CAAE:33396720.7.0000.5150) because all the data used were obtained through secondary databases that do not allow the identification of individuals.

## Author contributions

CR: writing—original draft, methodology, and visualization. MP: writing—review, editing, and viewing. IM: conceptualization, supervision, formal analysis, visualization, and writing—review and editing. AV: writing—review and editing, and software and original draft methodology. MM and AM: methodology and writing, review, and editing. All authors contributed to the article and approved the submitted version.

## Funding

This study was funded by the Conselho Nacional de Desenvolvimento e Científico e Tecnológico (CNPq/Brazil)—442636/2019-9; and Fundação de Amparo à Pesquisa do Estado de Minas Gerais (FAPEMIG/Brazil). This study was financed in part by the Coordenação de Aperfeiçoamento de Pessoal de Nível Superior—Brazil (CAPES)—Finance Code 001. This study used data from the Institute for Health Metrics and Evaluation (IHME), funded by Bill & Melinda Gates Foundation.

## Conflict of interest

The authors declare that the research was conducted in the absence of any commercial or financial relationship that could be interpreted as a potential conflict of interest.

## Publisher’s note

All claims expressed in this article are solely those of the authors and do not necessarily represent those of their affiliated organizations, or those of the publisher, the editors and the reviewers. Any product that may be evaluated in this article, or claim that may be made by its manufacturer, is not guaranteed or endorsed by the publisher.
