# Supplementary material for: Chronic diseases attributable to a diet rich in processed meat in Brazil: Burden and financial impact on the healthcare system
Source: Front Nutr. 2023 Mar 15;10:1114766. doi: 10.3389/fnut.2023.1114766 (PMC10050364; doi:10.3389/fnut.2023.1114766)
Supplement: Supplementary file 1 [file Data_Sheet_1.docx]

**Chronic diseases attributable to a diet rich in processed meat in Brazil: Burden and financial impact on the healthcare system**

**Table S1** Age-standardized disability-adjusted life years rates per 100,000 inhabitants for chronic noncommunicable diseases attributable to a diet rich in processed meat for both sexes and stratified by sex in Brazil between 1990 and 2019.

| **Year** | **Male** | **Female** | **Both** |
| --- | --- | --- | --- |
|  | **Age-standardized DALY rates per 100,000 inhabitants**  **(95% UI)** | **Age-standardized DALY rates per 100,000 inhabitants**  **(95% UI)** | **Age-standardized DALY rates per 100,000 inhabitants**  **(95% UI)** |
| 1990 | 80.59 (33.22–161.71) | 70.08 (34.00–120.19) | 75.31 (34.92–139.65) |
| 1991 | 77.62 (32.17–155.85) | 68.12 (33.51–116.07) | 72.85 (34.08–133.96) |
| 1992 | 76.22 (32.10–151.58) | 67.49 (33.04–114.13) | 71.85 (33.80–131.42) |
| 1993 | 76.47 (32.44–151.81) | 68.18 (33.66–114.62) | 72.32 (33.80–131.75) |
| 1994 | 74.94 (32.21–147.24) | 67.53 (33.23–113.24) | 71.25 (33.28–128.80) |
| 1995 | 73.49 (31.87–144.19) | 67.07 (33.17–111.47) | 70.33 (33.03–126.15) |
| 1996 | 72.79 (31.47–142.60) | 66.39 (32.97–109.96) | 69.63 (32.78–125.09) |
| 1997 | 71.25 (31.12–139.29) | 64.73 (32.26–107.18) | 68.02 (32.09–121.63) |
| 1998 | 71.08 (31.38–137.85) | 64.20 (32.20–105.41) | 67.64 (31.97–120.33) |
| 1999 | 70.78 (31.34–136.81) | 63.55 (31.92–103.86) | 67.14 (31.59–118.99) |
| 2000 | 70.35 (31.14–134.09) | 62.96 (31.87–102.19) | 66.61 (31.42–117.57) |
| 2001 | 70.38 (31.24–133.80) | 62.65 (31.68–101.80) | 66.46 (31.64–116.78) |
| 2002 | 70.83 (31.68–132.75) | 63.10 (32.27–101.59) | 66.90 (31.97–117.17) |
| 2003 | 72.06 (32.38–134.99) | 63.59 (32.90–102.54) | 67.73 (32.85–117.87) |
| 2004 | 73.52 (33.18–136.40) | 64.42 (33.52–103.50) | 68.85 (33.64–118.88) |
| 2005 | 73.41 (33.43–135.40) | 64.21 (33.85–101.89) | 68.69 (33.92–117.98) |
| 2006 | 74.67 (34.25–136.70) | 65.43 (35.10–103.91) | 69.92 (34.71–118.68) |
| 2007 | 76.34 (35.34–139.31) | 66.81 (36.23–104.96) | 71.43 (35.84–120.49) |
| 2008 | 78.45 (36.63–143.09) | 68.39 (37.47–105.87) | 73.25 (37.00–123.26) |
| 2009 | 80.29 (38.14–145.65) | 70.01 (38.88–108.90) | 74.97 (38.15–126.51) |
| 2010 | 81.95 (39.18–148.23) | 71.03 (39.83–109.69) | 76.29 (39.37–127.31) |
| 2011 | 83.65 (40.37–149.80) | 72.37 (40.65–111.66) | 77.80 (40.29–129.52) |
| 2012 | 84.31 (41.34–149.33) | 73.16 (41.35–111.42) | 78.54 (40.93–129.40) |
| 2013 | 85.55 (42.49–150.18) | 74.30 (42.02–112.67) | 79.73 (42.03–130.53) |
| 2014 | 86.05 (42.80–150.03) | 74.81 (42.34–113.23) | 80.24 (42.83–130.90) |
| 2015 | 86.50 (43.82–149.64) | 75.34 (42.97–114.22) | 80.72 (43.22–130.79) |
| 2016 | 88.36 (44.80–152.45) | 75.25 (42.95–113.29) | 81.52 (43.83–131.94) |
| 2017 | 87.43 (44.47–150.22) | 73.33 (42.08–110.42) | 80.05 (43.18–128.37) |
| 2018 | 86.87 (44.55–148.93) | 73.04 (42.08–110.17) | 79.65 (43.06–127.66) |
| 2019 | 86.47 (44.56–147.51) | 72.84 (41.52–109.83) | 79.35 (42.84–126.25) |

95% UI: 95% Uncertainty interval; DALY: disability-adjusted life year.

**Table S2** Age-standardized mortality rates per 100,000 inhabitants for chronic noncommunicable diseases attributable to a diet rich in processed meat for both sexes and stratified by sex in Brazil between 1990 and 2019.

| **Year** | **Male** | **Female** | **Both** |
| --- | --- | --- | --- |
|  | **Age-standardized mortality rates per 100,000 inhabitants**  **(95% UI)** | **Age-standardized mortality rates per 100,000 inhabitants**  **(95% UI)** | **Age-standardized mortality rates per 100,000 inhabitants**  **(95% UI)** |
| 1990 | 2.71(1.09–5.77) | 2.55 (1.19–4.69) | 2.64 (1.17–5.21) |
| 1991 | 2.60 (1.06–5.48) | 2.44 (1.16–4.47) | 2.53 (1.13–4.95) |
| 1992 | 2.55 (1.06–5.34) | 2.40 (1.13–4.35) | 2.49 (1.11–4.82) |
| 1993 | 2.57 (1.07–5.32) | 2.43 (1.15–4.38) | 2.51 (1.13–4.81) |
| 1994 | 2.51 (1.05–5.21) | 2.39 (1.14–4.34) | 2.46 (1.10–4.71) |
| 1995 | 2.44 (1.02–5.04) | 2.36 (1.12–4.27) | 2.41 (1.08–4.60) |
| 1996 | 2.41(1.02–4.93) | 2.33 (1.11–4.18) | 2.38 (1.07–4.56) |
| 1997 | 2.34 (1.00–4.80) | 2.26 (1.07–4.02) | 2.31 (1.05–4.41) |
| 1998 | 2.34 (1.00–4.76) | 2.24 (1.07–3.93) | 2.30 (1.04–4.32) |
| 1999 | 2.33 (0.99–4.67) | 2.20 (1.06–3.88) | 2.27 (1.03–4.26) |
| 2000 | 2.31 (0.99–4.64) | 2.17 (1.06–3.80) | 2.25 (1.03–4.18) |
| 2001 | 2.30 (0.99–4.58) | 2.15 (1.05–3.74) | 2.23 (1.03–4.12) |
| 2002 | 2.31 (1.00–4.54) | 2.15 (1.06–3.73) | 2.23 (1.04–4.10) |
| 2003 | 2.33 (1.02–4.56) | 2.15 (1.05–3.70) | 2.24 (1.05–4.09) |
| 2004 | 2.36 (1.04–4.58) | 2.15 (1.07–3.70) | 2.26 (1.06–4.11) |
| 2005 | 2.33 (1.01–4.52) | 2.11 (1.05–3.61) | 2.22 (1.05–4.01) |
| 2006 | 2.36 (1.03–4.55) | 2.14 (1.06–3.63) | 2.25 (1.05–4.07) |
| 2007 | 2.40 (1.05–4.61) | 2.17 (1.10–3.68) | 2.29 (1.08–4.14) |
| 2008 | 2.46 (1.08–4.71) | 2.20 (1.12–3.71) | 2.33 (1.10–4.20) |
| 2009 | 2.51(1.11–4.82) | 2.24 (1.14–3.77) | 2.38 (1.14–4.30) |
| 2010 | 2.56 (1.15–4.86) | 2.26 (1.17–3.79) | 2.41 (1.17–4.35) |
| 2011 | 2.60 (1.18–4.93) | 2.29 (1.20–3.83) | 2.44 (1.19–4.36) |
| 2012 | 2.59 (1.18–4.87) | 2.27 (1.18–3.75) | 2.43 (1.20–4.29) |
| 2013 | 2.61 (1.20–4.93) | 2.27(1.19–3.75) | 2.44 (1.21–4.32) |
| 2014 | 2.60 (1.21–4.86) | 2.25 (1.18–3.72) | 2.43 (1.22–4.28) |
| 2015 | 2.60 (1.21–4.86) | 2.25 (1.20–3.71) | 2.43 (1.22–4.26) |
| 2016 | 2.64 (1.25–4.94) | 2.26 (1.20–3.67) | 2.45 (1.24–4.29) |
| 2017 | 2.57 (1.21–4.74) | 2.20 (1.18–3.59) | 2.38 (1.21–4.18) |
| 2018 | 2.54 (1.22–4.70) | 2.19 (1.17–3.61) | 2.37 (1.20–4.14) |
| 2019 | 2.54 (1.24–4.69) | 2.18 (1.16–3.53) | 2.36 (1.23–4.09) |

95% UI: 95% Uncertainty interval.

**Table S3** Age-standardized disability-adjusted life years and deaths rates per 100,000 inhabitants for chronic noncommunicable diseases attributable to a diet rich in processed meat for both sexes in the federative units of Brazil in 1990 and 2019.

| **Federative unit** | **Age-standardized mortality rates per 100,000 inhabitants (95% UI)** | | **Age-standardized DALY rates per 100,000 inhabitants (95% UI)** | |
| --- | --- | --- | --- | --- |
|  | **1990** | **2019** | **1990** | **2019** |
| Acre | 1.86 (0.84–3.56) | 2.10 (1.07–3.57) | 51.73 (24.56–92.45) | 70.84 (37.36–109–61) |
| Alagoas | 2.67 (1.24–4.81) | 3.47 (1.77–5.65) | 76.76 (35.74–130.45) | 111.20 (58.26–173,61) |
| Amapá | 1.66 (0.73–3.28) | 1.99 (1.03–3.42) | 48.26 (22.67–86.91) | 69.84 (37.78–107.93) |
| Amazonas | 2.16 (0.96–4.24) | 2.16 (1.16–3.51) | 59.33 (28.30–105.72) | 73.98 (41.80–110.15) |
| Bahia | 2.26 (1.03–4.15) | 2.65 (1.36–4.32) | 66.98 (30.39–117.81) | 86.93 (45.65–135.94) |
| Ceará | 1.55 (0.68–3.00) | 2.16 (1.06–3.86) | 45.29 (20.52–82.72) | 70.55 (37.21–114.95) |
| Distrito Federal | 3.30 (1.51–6.46) | 2.28 (1.15–3.88) | 85.50 (40.27–154.09) | 77.20 (42.59–118.79) |
| Espírito Santo | 2.27 (0.97–4.56) | 2.47 (1.23–4.45) | 63.77 (28.52–118.83) | 82.62 (43.29–134.63) |
| Goiás | 2.28 (0.98–4.68) | 2.22 (1.06–4.05) | 65.06 (28.82–124.52) | 76.48 (39.44–127.16) |
| Maranhão | 2.40 (1.04–4.60) | 3.22 (1.61–5.67) | 73.23 (31.27–138.68) | 99.47 (51.49–165.64) |
| Mato Grosso | 2.16 (0.92–4.51) | 2.23 (1.12–3.77) | 60.95 (26.37–118.23) | 76.78 (41.14–118.97) |
| Mato Grosso do Sul | 2.30 (0.93–4.88) | 2.14 (1.03–4.04) | 67.13 (29.22–128.43) | 77.40 (41.11–128.91) |
| Minas Gerais | 2.52 (1.04–5.12) | 1.84 (0.92–3.29) | 70.05 (30.25–136.20) | 64.88 (34.03–106.82) |
| Pará | 2.07 (0.92–4.20) | 2.28 (1.17–3.86) | 57.26 (25.34–106.78) | 76.58 (41.78–122.15) |
| Paraíba | 2.17 (1.01–3.99) | 2.70 (1.34–4.59) | 62.24 (27.78–109.78) | 89.00 (45.90–141.32) |
| Paraná | 2.57 (1.06–5.37) | 2.35 (1.18–4.20) | 69.37 (30.47–136.14) | 78.10 (41.46–125.90) |
| Pernambuco | 2.70 (1.20–5.12) | 3.22 (1.64–5.61) | 76.02 (34.31–135.81) | 100.74 (53.51–164.16) |
| Piauí | 2.00 (0.88–3.72) | 2.16 (1.05–3.69) | 54.71 (24.16–98.56) | 73.74 (39.16–116.94) |
| Rio de Janeiro | 3.69 (1.59–7.34) | 2.92 (1.45–5.26) | 105.69 (48.97–201.30) | 97.50 (51.29–162.11) |
| Rio Grande do Norte | 2.11 (0.96–4.01) | 2.62 (1.29–4.48) | 60.61 (29.06–107.59) | 86.70 (45.92–137.84) |
| Rio Grande do Sul | 2.50 (1.05–5.23) | 2.19 (1.11–3.87) | 68.79 (30.36–136.83) | 71.43 (37.68–114.35) |
| Rondônia | 2.98 (1.29–5.90) | 2.48 (1.24–4.28) | 75.85 (33.94–139.29) | 81.61 (42.79–132.02) |
| Roraima | 2.92 (1.34–5.35) | 2.80 (1.48–4.44) | 77.05 (36.38–134.02) | 88.92 (49.36–133.92) |
| Santa Catarina | 2.39 (1.01–4.89) | 2.05 (1.05–3.64) | 63.44 (26.35–121.08) | 66.39 (35.68–108.54) |
| São Paulo | 3.17 (1.35–6.66) | 2.12 (1.02–4.03) | 88.56 (40.67–170.27) | 74.74 (40.01–125.01) |
| Sergipe | 2.89 (1.36–4.93) | 2.88 (1.45–4.70) | 76.49 (36.34–125.28) | 93.03 (49.75–146.00) |
| Tocantins | 2.16 (0.96–4.19) | 2.63 (1.32–4.53) | 55.83 (25.03–101.90) | 83.79 (44.55–134.05) |

95% UI: 95% Uncertainty interval; DALY: disability-adjusted life year.

**Table S4** Age-standardized disability-adjusted life years and deaths rates per 100,000 inhabitants according to specific causes of chronic noncommunicable diseases attributable to a diet rich in processed meat for both sexes in Brazil in 2019.

| **Outcome** | **Age-standardized mortality rates per 100,000 inhabitants (95% UI)** | **Age-standardized DALY rates per 100,000 inhabitants (95% UI)** |
| --- | --- | --- |
| Type 2 diabetes mellitus | 1.22 (0.67–1.52) | 50.75 (28.29–67.27) |
| Ischemic heart disease | 0.92 (0.16–2.66) | 23.35 (3.35–67.43) |
| Colorectal cancer | 0.21 (0.03–0.36) | 5.26 (0.67–9.05) |

95% UI: 95% Uncertainty interval; DALY: disability-adjusted life year.

**Table S5** Total costs of hospitalizations and outpatient procedures for chronic noncommunicable diseases attributable to a diet rich in processed meats, to the Unified Health System in the Brazilian federative units in 2019.

| **Federative unit** | **Costs to the SUS (R$)** | | | **Costs for SUS (US$)** | | |
| --- | --- | --- | --- | --- | --- | --- |
|  | **Outpatient** | **Hospitalization** | **Total** | **Outpatient** | **Hospitalization** | **Total** |
| Acre | 7,435.56 | 32,360.66 | 39,796.22 | 1,885.28 | 8,205.04 | 10,090.32 |
| Alagoas | 107,266.83 | 217,549.70 | 324,816.53 | 27,197.47 | 55,159.66 | 82,357.13 |
| Amapá | 16,260.24 | 48,040.19 | 64,300.43 | 4,122.78 | 12,180.58 | 16,303.36 |
| Amazonas | 64,076.62 | 216,358.20 | 280,434.82 | 16,246.61 | 54,857.56 | 71,104.17 |
| Bahia | 397,189.04 | 1,057,209.45 | 1,454,398.49 | 100,707.16 | 268,055.13 | 368,762.29 |
| Ceará | 198,072.85 | 835,795.62 | 1,033,868.47 | 50,221.31 | 211,915.73 | 262,137.04 |
| Distrito Federal | 108,098.15 | 345,987.19 | 454,085.34 | 27,408.25 | 87,724.95 | 115,133.20 |
| Espírito Santo | 253,073.92 | 706,260.17 | 959,334.09 | 64,166.81 | 179,072.05 | 243,238.86 |
| Goiás | 304,005.52 | 748,255.47 | 1,052,260.99 | 77,080.51 | 189,719.95 | 266,800.46 |
| Maranhão | 97,186.91 | 206,588.85 | 303,775.76 | 24,641.71 | 52,380.54 | 77,022.25 |
| Mato Grosso | 115,122.28 | 301,825.44 | 416,947.72 | 29,189.22 | 76,527.75 | 105,716.97 |
| Mato Grosso do Sul | 114,588.98 | 457,605.79 | 572,194.77 | 29,054.00 | 116,025.81 | 145,079.81 |
| Minas Gerais | 1,177,385.69 | 3,552,487.09 | 4,729,872.78 | 298,525.78 | 900,732.02 | 1,199,257.80 |
| Pará | 80,591.97 | 277,173.78 | 357,765.75 | 20,434.07 | 70,277.33 | 90,711.40 |
| Paraíba | 93,126.48 | 330,126.25 | 423,252.73 | 23,612.19 | 83,703.41 | 107,315.60 |
| Paraná | 921,429.99 | 3,641,406.02 | 4,562,836.01 | 233,628.29 | 923,277.39 | 1,156,905.68 |
| Pernambuco | 282,248.94 | 889,495.68 | 1,171,744.62 | 71,564.13 | 225,531.36 | 297,095.49 |
| Piauí | 74,017.02 | 166,660.30 | 240,677.32 | 18,766.99 | 42,256.67 | 61,023.66 |
| Rio de Janeiro | 757,675.74 | 1,770,541.59 | 2,528,217.33 | 192,108.45 | 448,920.28 | 641,028.73 |
| Rio Grande do Norte | 148,541.54 | 443,733.08 | 592,274.62 | 37,662.66 | 112,508.39 | 150,171.05 |
| Rio Grande do Sul | 874,519.33 | 2,283,883.65 | 3,158,402.98 | 221,734.11 | 579,078.00 | 800,812.11 |
| Rondônia | 46,852.46 | 114,032.62 | 160,885.08 | 11,879.43 | 28,912.94 | 40,792.37 |
| Roraima | 1,627.62 | 36,213.49 | 37,841.11 | 412.68 | 9,181.92 | 9,594.60 |
| Santa Catarina | 453,355.85 | 1,590,974.21 | 2,044,330.06 | 114,948.24 | 403,391.03 | 518,339.27 |
| São Paulo | 2,910,114.03 | 6,793,322.70 | 9,703,436.73 | 737,858.53 | 1,722,444.90 | 2,460,303.43 |
| Sergipe | 34,684.45 | 167,524.65 | 202,209.10 | 8,794.23 | 42,475.82 | 51,270.05 |
| Tocantins | 36,505.26 | 103,811.73 | 140,316.99 | 9,255.90 | 26,321.43 | 35,577.33 |

SUS: Unified Health System.

**Table S6** Costs of hospitalizations and outpatient procedures per 10,000 inhabitants for chronic noncommunicable diseases attributable to a diet rich in processed meats to the Unified Health System in the Brazilian federative units in 2019.

| **Federative unit** | **Costs US$ per 10,000 inhabitants** |
| --- | --- |
| Acre | 235.79 |
| Alagoas | 428.26 |
| Amapá | 394.13 |
| Amazonas | 330.65 |
| Bahia | 389.01 |
| Ceará | 481.90 |
| Distrito Federal | 575.55 |
| Espírito Santo | 930.48 |
| Goiás | 617.58 |
| Maranhão | 203.50 |
| Mato Grosso | 511.60 |
| Mato Grosso do Sul | 857.10 |
| Minas Gerais | 864.04 |
| Pará | 196.57 |
| Paraíba | 434.26 |
| Paraná | 1,564.06 |
| Pernambuco | 514.54 |
| Piauí | 318.83 |
| Rio de Janeiro | 574.05 |
| Rio Grande do Norte | 689.87 |
| Rio Grande do Sul | 1,046.32 |
| Rondônia | 374.41 |
| Roraima | 349.73 |
| Santa Catarina | 1,107.59 |
| São Paulo | 818.63 |
| Sergipe | 374.66 |
| Tocantins | 395.94 |
